# Urologic malignancies: advances in the analysis and interpretation of clinical findings

**DOI:** 10.2144/fsoa-2020-0210

**Published:** 2021-02-04

**Authors:** Felice Crocetto, Carlo Buonerba, Vincenzo Caputo, Matteo Ferro, Francesco Persico, Francesco Trama, Ester Iliano, Sebastiano Rapisarda, Maida Bada, Gaetano Facchini, Antonio Verde, Sabino De Placido, Biagio Barone

**Affiliations:** 1 Department of Neurosciences, Human Reproduction & Odontostomatology, University of Naples Federico II, Naples 80131, Italy; 2 Department of Oncology & Hematology, Regional Reference Center for Rare Tumors, AOU Federico II of Naples, Naples 80131, Italy; 3 Division of Urology, European Institute of Oncology-IRCCS, Milan 20122, Italy; 4 Centro di Referenza Nazionale per l'Analisi e Studio di Correlazione tra Ambiente, Animale e Uomo, Istituto Zooprofilattico Sperimentale del Mezzogiorno, Portici, Naples 80055, Italy; 5 Department of Urology, Humanitas Clinical & Research Center-IRCCS, Rozzano, Milan 20089, Italy; 6 Andrology & Urogynecological Clinic, Santa Maria Terni Hospital, University of Perugia, Terni 05100, Italy; 7 Department of Urology, Ospedale Pederzoli, Peschiera del Garda, Verona 37019, Italy; 8 Department of Urology, San Bassiano Hospital, Bassano del Grappa, Vicenza 36061, Italy; 9 UOC of Medical Oncology, ASL NA 2 Nord, “S M delle Grazie” Hospital, Pozzuoli, Naples 80078, Italy; 10 Department of Clinical Medicine & Surgery, University Federico II of Naples, Naples 80131, Italy

**Keywords:** biomarkers, immunotherapy, urologic malignancies

The most commonly occurring urologic malignancies include prostate, bladder and kidney cancer. While prostate cancer is diagnosed in males, bladder and kidney cancer are more commonly reported in males versus females. In male US citizens, prostate, bladder and kidney cancer account for 191,930, 62,100 and 45,520 cases, respectively, of the approximately 893,660 new cancer cases expected in 2020. In the US women, of 912,930 new cancer cases estimated to be diagnosed in 2020, bladder and kidney cancer account for 19,300 and 28,230 cases, respectively. Interestingly, 5-year survival rates in localized disease are >60% in prostate and kidney cancer but remain unsatisfactory in bladder cancer (≈30%), while also remaining disappointing in patients with distant metastasis (≈5% in prostate and bladder cancer, ≈15% in kidney cancer); this underlines the compelling need for more effective systemic therapeutic options [1].

In prostate cancer, over the past decade, advances in systemic therapy have not been mostly accomplished by introducing novel agents [2], but rather by developing novel indications for approved treatments [3]. Large, randomized Phase III trials have demonstrated that men with metastatic, castration-sensitive prostate cancer can benefit from both docetaxel and novel hormonal agents, including androgen-receptor-axis-target agents (ARAT) such as enzalutamide, abiraterone and apalutamide [4]. A recently conducted network meta-analysis showed that while men with castration-sensitive prostate cancer treated with either docetaxel or an ARAT agent as first-line therapy reported an overall hazard ratio (HR) for death of 0.69 (95% CI: 0.61–0.78), those treated with an ARAT agent versus docetaxel showed a HR for death favoring the ARAT group of 0.78 (95% CI: 0.67–0.91) [4]. Conversely, sequential or concomitant use of docetaxel plus an ARAT agent in the castration-sensitive setting is not expected to provide any additional benefit compared with the use of an ARAT agent alone [5]. Significant progresses have been made with the results obtained with the poly(ADP-ribose) polymerase (PARP) inhibitor olaparib, which was capable of prolonging survival in selected men with mutations in *BRCA1*, *BRCA2* or *ATM* genes [6]. In the cohort of the PROFOUND trial including 245 men with at least one alteration in *BRCA1*, *BRCA2* or *ATM*, olaparib compared with physician’s choice of enzalutamide or abiraterone was associated with a significantly longer survival, with a HR for death of 0.69; 95% CI: 0.50–0.97; p = 0.02 (median overall survival [OS], 19.1 months with olaparib and 14.7 months with control therapy). Although these results represent a breakthrough in prostate cancer, it must be noted that only a minority of patients with advanced prostate cancer harbor these mutations, and additional research is needed to explore whether olaparib-based combination therapy may be effective in unselected men.

In bladder cancer, the advent of immunotherapy based on antiprogrammed death 1(PD-1)/programmed death ligand-1 (PDL-1) inhibitors has provided a survival advantage in patients with advanced cancer who have a poor prognosis. In the JAVELIN Bladder 100 trial, patients with advanced urothelial cancer were randomized to best supportive care with or without the anti-PDL-1 agent avelumab after completing the primary platinum-based systemic treatment. In the study cohort consisting of 700 patients, 1-year OS rate was 71.3 in the avelumab group and 58.4% in the control group, with a HR for death of 0.69 (95% CI: 0.56–0.86; p = 0.001) [7]. These results are consistent with those obtained in the randomized Phase III trial KEYNOTE 045, in which 542 patients with recurrent advanced urothelial cancer that recurred or progressed after platinum-based chemotherapy were randomized to anti PD-1 agent pembrolizumab at a dose of 200 mg every 3 weeks or the investigator’s choice of chemotherapy with vinflunine, paclitaxel or docetaxel. In the overall cohort, median OS was 10.3 versus 7.4 months in the pembrolizumab versus chemotherapy group (HR for death: 0.73; 95% CI: 0.59–0.91; p = 0.002). Although survival results seem to be slightly more favorable in the JAVELIN trial, additional studies are required to establish the optimal approach to deliver anti PD-1/PDL-1 therapy (at the end of first-line platinum chemotherapy versus at progression after first-line platinum chemotherapy).

In kidney cancer, significant progress has been made with the results obtained with anti PD-1/PDL-1 agents in the first-line setting. Avelumab, pembrolizumab, nivolumab and atezolizumab have been successfully combined with both anti-VEGF (avelumab + axitinib, pembrolizumab+ axitinib, atezolizumab + bevacizumab) and anti-CTLA-4 agents (nivolumab + ipilimumab) [8]. Importantly, pembrolizumab + axitinib in unselected patients and ipilimumab + nivolumab in patients at intermediate/poor prognosis are recommended by European Society of Medical Oncology guidelines (ESMO) [9]. Importantly, the results from the Phase III CheckMate 9ER study assessing nivolumab plus cabozantinib versus sunitinib in the first-line treatment setting of advanced clear cell renal cell carcinoma (RCC) were recently presented at the ESMO 2020 Virtual Congress [10]. In this trial, 651 patients were randomized in a 1:1 ratio to receive a 240 mg flat dose of iv. nivolumab every 2 weeks plus oral cabozantinib at 40 mg once daily or oral sunitinib at 50 mg for 4 weeks in 6-week cycles. Of note, with median follow-up of 18.1 months, a median progression-free survival of 16.6 versus 8.3 months (HR: 0.51; 95% CI: 0.41–0.64; p < 0.0001) was achieved, with a HR of 0.60 (98.89% CI: 0.40–0.89; p = 0.0010. These results led ESMO guidelines to include combination of cabozantinib + nivolumab among the recommended first-line therapies for advanced clear cell RCC [9].

The wealth of effective treatments, which are approved on an ‘average’ efficacy, underlines the compelling need for additional predictors of efficacy and also toxicity to incorporate in the therapeutic algorithm. In fact, trials show how there is a substantial proportion of patients with urological malignancies who do not benefit at all from the novel treatments discussed. Such a need can be satisfied by three different research tools, which include: prospective and retrospective, nontranslational clinical studies; prospective and retrospective, biomarker-based observational clinical studies; meta-analysis of randomized-controlled trials assessing the influence of baseline clinical variables and biomarkers on treatment efficacy.

In prostate cancer, our work group conducted two observational studies in patients treated with the taxane agent cabazitaxel after failure of docetaxel and showed that a higher Gleason score was associated with improved efficacy of cabazitaxel [11] and that baseline neutrophil count of less than 4570/mm was associated with increased risk of severe neutropenia [12]. Also, meta-analysis of published trials on the use of ARAT agents in the metastatic castration-sensitive setting showed that a high tumor volume according to the CHAARTED criteria and presence of visceral metastasis were associated with decreased progression-free survival outcomes [5].

In patients with kidney cancer, a recently published meta-analysis showed that therapy with anti PD-1/PDL-1 agents was associated with a significantly greater overall and complete response rate in PD-L1 positive versus negative patients (odds ratio (OR): 1.84; 95% CI: 1.48–2.28; OR: 3.11; 95% CI: 2.04–4.75) [8]. Another recently published meta-analysis conducted by our work group assessed data from 3814 RCC patients receiving anti PD-1/PDL-1 based combination therapy in the intention-to-treat cohort, of whom 512 showed sarcomatoid features. In the sarcomatoid RCC subgroup versus the ITT population, pooled estimates of the HR for progression or death were 0.57 (95% CI: 0.45–0.74) versus 0.79 (95% CI: 0.70–0.89), respectively, with a statistically significant interaction favoring patients with sarcomatoid RCC. Furthermore, patients with versus without sarcomatoid features reported a higher increase in complete response rate +0.10 (95% CI: 0.04–0.16) versus +0.04 (95% CI: 0.00–0.07), with a statistically significant difference of +0.06 (95% CI: 0.02–0.10; p = 0.007) [13].

Finally, in bladder cancer the role of PD-L1 expression in predicting the benefit from anti PD-1/PDL-1 agents is unclear [14], although molecular subtypes of bladder cancers may provide significant prognostic and predictive information [15].

In conclusion, significant progress has expanded the therapeutic scenario over the last year in urologic malignancies. The need for effective biomarkers necessary to tailor treatment can be satisfied by appropriately designed clinical studies and meta-analysis. In this regard, harmonization of baseline variables (e.g., cutoff values for PDL-1 expression) is also essential to pool data from different randomized-controlled trials.
